# Is There a Clinical Benefit of Abdominal Binders After Abdominal Surgery: A Systematic Literature Review

**DOI:** 10.3389/jaws.2024.13506

**Published:** 2024-10-17

**Authors:** Nicolas Michot, Pablo Ortega-Deballon, Elias Karam, Urs Pabst-Giger, Mehdi Ouaissi

**Affiliations:** ^1^ Department of Digestive, Oncological, Endocrine, Hepato-Biliary, Pancreatic Surgery and Liver Transplantation, Trousseau Hospital, Tours University Hospital, Chambray-Lès-Tours, France; ^2^ Department of Digestive Surgery, François Mitterand Hospital, Dijon University Hospital, Dijon, France; ^3^ Fliedner Fachhochschule, University of Applied Sciences Düsseldorf, Düsseldorf, Germany

**Keywords:** abdominal binders, laparotomy, abdominal surgery, postoperative complications, postoperative pain

## Abstract

**Background:**

The incidence of incisional hernia following laparotomy varies between 2% and 30%. It is well-established that the need to control several risk factors prior to surgery exists (weight loss before surgery, diabetes control). Postoperative abdominal binder (AB) is often recommended by surgeons, yet evidence on this topic is lacking. The aim of this review was to present current evidence on the use of abdominal binders after abdominal surgery.

**Material and Methods:**

A comprehensive literature review between January and May 2024 was conducted using a range of search engines, including PubMed, Science Direct, EMBASE, Google Scholar, and Google. The following keywords were used: “abdominal binder,” “abdominal support,” “hernia,” “girdle and hernia,” “compression belt and hernia,” and “abdominal support and hernia.”

**Results:**

Sixteen articles were selected for further analysis (7 RCTs, 6 non-RCTs and 3 meta-analyses). None of the studies reported a reduction in the incidence of abdominal dehiscence or incisional hernia. Postoperative use of the AB has been shown to reduce postoperative discomfort and pain for a limited period of up to 48–72 h. There was no discernible difference in the incidence of surgical site complications.

**Conclusion:**

The current evidence indicates that the use of AB following abdominal surgery is safe, although no benefit has been established (except 48 h after surgery). AB may enhance comfort in select patients; however, further studies are necessary to justify their routine use, with a particular focus on the medical and economic implications.

## Introduction

The incidence of incisional hernia following laparotomy has been reported to range from 2% to 30% in various studies [1–6]. The risk of recurrence after incisional hernia repair in large series is between 25% and 63% [2, 5, 7, 8]. This complication is most frequently observed between 18 and 24 months following the initial surgery [4, 5]. The risk of postoperative hernia is significantly increased by age, diabetes, obesity, smoking, length of the laparotomy size and the onset of surgical site infection significantly increase the risk of postoperative hernia [5, 6]. In France, the French Surgical Association (AFC) reports that 45,242 incisional hernia treatments were performed in 2017. The estimated annual national cost, inclusive of both public and private treatment, is 172 million euros. In light of the significant health burden associated with incisional hernia, prevention and treatment remain a crucial public health concern [9].

A number of preventive measures have been demonstrated to be effective in clinical studies, including weight loss prior to surgery and the management of diabetes [10]. However, the efficacy of other proposed preventive measures remains a topic of debate. Amongst these, the use of abdominal binders (AB) after surgery appears to be an intuitive method of preventing excess abdominal tension in the postoperative period. In 2014, a questionnaire was distributed to a number of French centres specializing in digestive surgery (50 responding centres). A total of 94% of the surgical teams prescribed an AB with the expectation that it would reduce the risk of incisional hernia by 83% [11]. There are few prospective randomized trials that have examined the possible effect of AB on the incidence of postoperative pain, wound complications, or incisional hernias. These trials have relatively small numbers of participants overall, and the procedures performed in the trials vary widely from general surgery to laparoscopic inguinal hernia repair to caesarean section [12–18]. In addition, none of the trials evaluated a possible preventive effect of wearing an AB on the incidence of postoperative incisional hernias after laparotomy as a primary endpoint. Despite the lack of scientific evidence supporting the preventive postoperative use of an AB, many general surgeons prescribe an AB as part of their clinical routine practice. The objective of this review was to ascertain the current evidence regarding the potential clinical benefits of AB after surgery.

## Material and Methods

### Selection of Articles

A comprehensive literature review was conducted using a range of search engines, including PubMed, Science Direct, EMBASE, Google Scholar, and Google. The following keywords were used: The following search terms were used: “abdominal binder,” “abdominal support,” “hernia,” “girdle and hernia,” “compression belts and hernia,” and “abdominal support and hernia.” All the results were then subjected to a second round of review by two senior surgeons (MO, NM) at our centre, who selected the articles that were deemed to be consistent with the review that we were undertaking. The updated PRISMA guidelines for the reporting of systematic reviews was applied [19]. Only literature from the year 2000 onwards was considered, because at that time the technique of abdominal wall closure was optimized by the use of slow absorbable continuous sutures [20]. Inclusion criteria for our systematic review include publications published between 2000 and 2023 that provide detailed information on the use of postoperative abdominal wall dressings after various abdominal procedures (digestive tract, gynecology, urology). The procedures for wearing an AB had to be detailed, including the duration of wear, the period of time it was to be worn during the day (all day, night, day, during exercise), and the effects of wearing the AB, including the occurrence of postoperative complications and recurrence. Exclusion criteria: cases not involving humans, case reports, articles that only specify the wearing of an AB without defining the stipulations for wearing the AB or explaining the consequences of the procedure (postoperative complications and/or recurrence), articles about wearing the AB in exceptional situations (post-accident abdominal trauma) where surgery had not been performed.

### Risk of Bias and Quality Assessment

Two experienced surgeons (MO, NM) independently assessed all articles using the ROBINS-I tool [21] for non-randomized controlled trials and the GRADE score [22].

## Results

**FIGURE 1:**
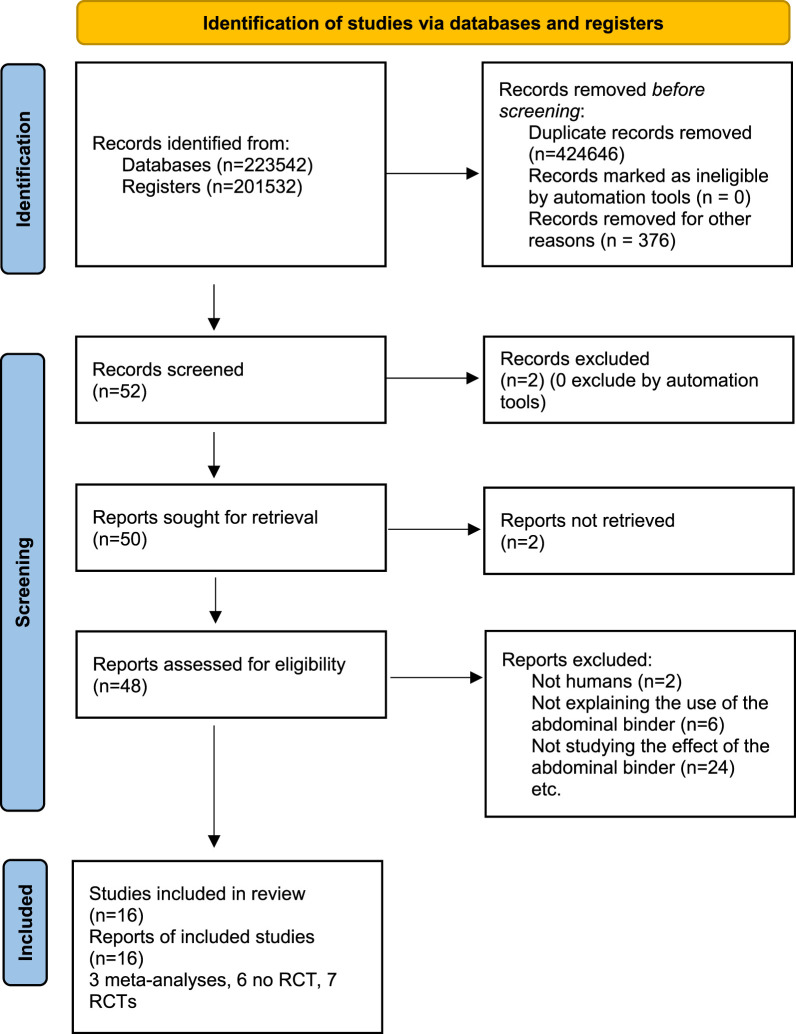
PRISMA 2020 flow diagram. RCT = Randomised controlled trial.

**FIGURE 2:**
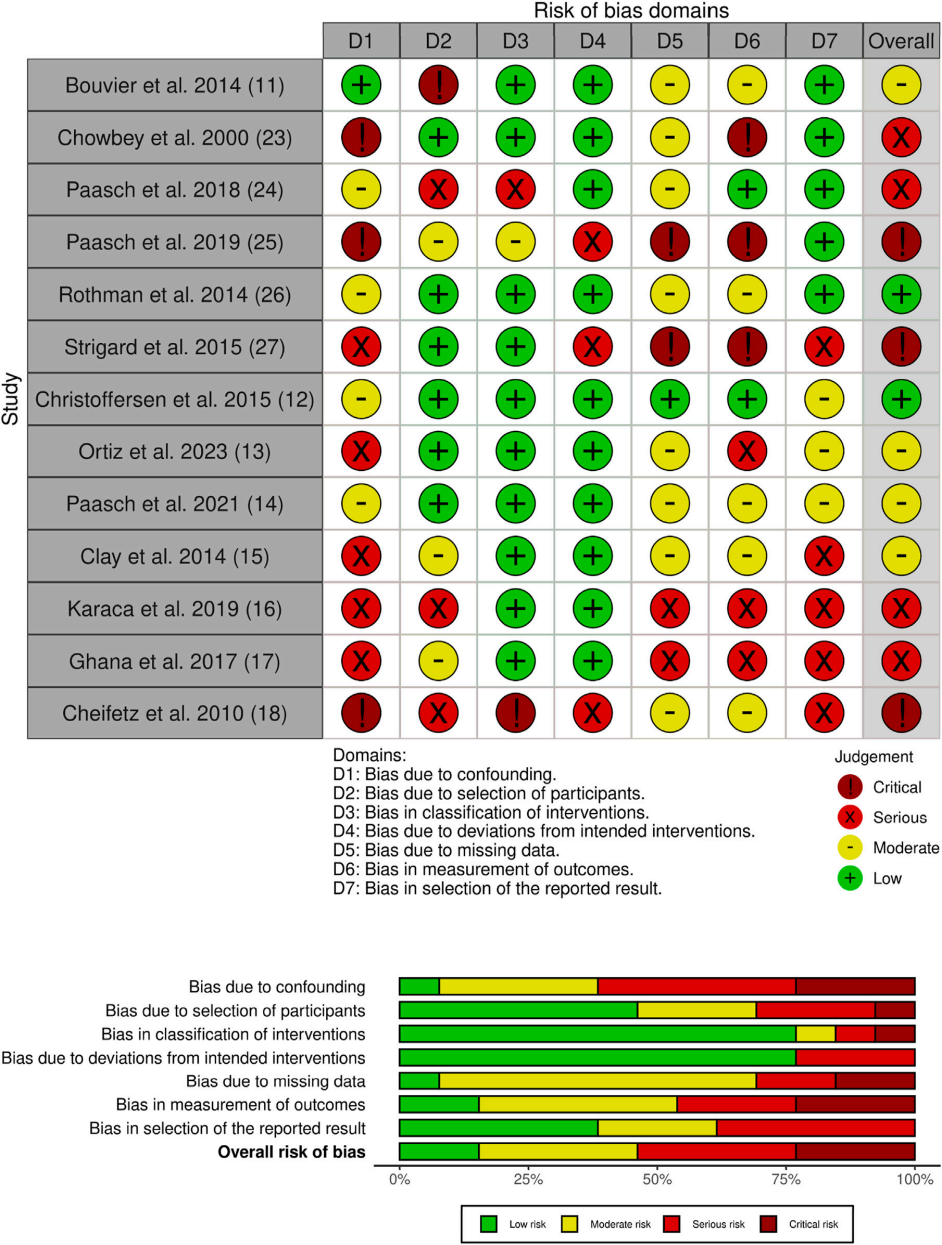
ROBINS-I tool for risk of bias assessment.

A total of 52 articles were identified between January and May 2024. After excluding irrelevant articles, sixteen articles were selected for further analysis [11–18, 23–30]. These included a total of seven randomized controlled trials (RCTs), 6 no RCT and 3 meta-analyses (Figure 1 flow charts) (Figure 2). Details of the RCTs selection are shown in Table 1. We therefore identified the articles that primarily investigate the use of AB through questionnaires. In the literature, only two articles are reported (Table 2), which consisted of sending questionnaires to German and French surgical teams asking about the prescription guidance for AB postoperatively. The French study, the only one to analyse the guidance for post-operative prescription with a preventive objective after a first laparotomy, and the second study, which related to the prescription for wearing an AB after surgery for an incisional hernia, were the only ones to address this topic. A first French study from 2014 by Bouvier et al. [11] obtained responses from 50 centres. Among the respondents, 31.9% indicated that they prescribed the AB with the objective of reducing post-operative complications, such as evisceration or incisional hernia. 14.9% of respondents stated that they prescribed the AB for the patient’s comfort, while the remaining respondents indicated that they prescribed it for both reasons. In this study, 40.4% of respondents indicated that they would prescribe the AB following incisional hernia treatment, 25.5% indicated that they would prescribe the AB for all laparotomies, and 17% indicated that they would prescribe it according to the size of the laparotomies. With regard to the duration of prescription, the AB was prescribed for a period of 1 week in 2% of centers, for a period of 1 month in 48.9% of cases, and for a period of 2 to 3 months in 31.9% of cases. The study by Paasch et al. [24] evaluated the various postoperative prescriptions following incisional hernia. Of the 44 centers that responded, 4.45% of teams prescribed the AB for 1 week, 15.9% for 15 days, 2.27% for 40 weeks, and 29.5% did not prescribe one. The AB was to be worn continuously throughout the day, including during exercise. These studies demonstrate that the durations and prescription guidance vary considerably depending on the surgeons and the different centres surveyed. Moreover, the prescriptions appear to be more influenced by the centre’s own common practice than by scientific evidence.

**TABLE 1:** GRADE evidence profile for randomized controlled trial.

Study	Surgical intervention	Risk of bias	Principal objective was compared in study	Inconsistency	Indirectness	Imprecision	Publication bias	Summary of findings		Results	Quality
								AB	Control		
Christoffersden et al. [12]	Post parietal repair	Not serious	Pain/seroma	Not serious	Not serious	Serious	Not serious	28	28	No clinical effect	Moderate
Ortiz et al. [13]	Post parietal surgery	Very serious	Post operative complication (included post operative hernia)	Serious	Serious	Serious	Not serious	21	19	No clinical effect	Very low
Paasch et al. [14]	Post parietal surgery	Not serious	Utility of AB	Not serious	Not serious	Serious	Not serious	18	19	IC95 [1; 35] *p* = 0.042	Moderate
Clay et al. [15]	Post colorectal surgery	Serious	Respiratory function	Serious	Serious	Serious	Not serious	23	25	No clinical effect	Very low
Karaca et al. [16]	Post cesarien	Serious	Post operative physical function	Not serious	Not serious	Not serious	Not serious	45	44	*p* = 0.001	Moderate
Ghana et al. [17]	Post cesarien	Serious	Post operative abdominal pain	Serious	Serious	Serious	Not serious	89	89	*p* < 0.001	Very low
Cheifetz et al. [18]	Post abdominal surgery	Very serious	Post operative physical function	Very serious	Very serious	Very serious	Not serious	30	30	*p* < 0.001	Very low

AB, abdominal binder.

**TABLE 2:** Guidance for wearing the abdominal binder (AB).

Retrospective Questionnaire Study	Population Analysed Digestive surgeon	Duration of AB prescription	Prescription guidance	Postoperative guidance
Bouvier et al. [11]	French multicenter study with 50 centres Analysis of AB prescriptions after all laparotomies	1 month: 48.9% 2–3 months: 31.9% 1 week: 2%	Reduce postoperative complications 31.9% (evisceration, incisional hernia) Patient comfort: 14.9% For the 2 reasons: 51.1%	All laparotomies: 25.5% Depending on the size of the laparotomy: 17% After incisional hernia treatment: 40.4%
Paasch et al. [14]	German Centre 44 centres Analysis of AB prescriptions after hernia treatment	1 week: 4.45% 15 days: 15.9% 40 weeks: 2.27% No AB prescription: 40%	Prevention of recurrence of hernia	Questionnaire relating to incisional hernia patients

The analysis of the literature focused on the results relating to pain and the occurrence of seroma in the postoperative period, depending on the use or non-use of an AB.

### Post-Operative Pain (Table 3)

Most studies showed no difference in postoperative pain between patients who wore an AB and those who did not. Only 2 randomized articles with 37 and 48 patients, respectively, showed an improvement in pain relief at postoperative day (POD) 2 and POD 5 [15, 25]. In these two studies, pain was assessed using the patient’s visual analog scale (VAS). The first study including 48 patients found that AB had no effect on pulmonary function, but that pain was significantly less in the AB group on POD 5. The second smaller study by Paash et al. reported significantly less postoperative pain in the AB group after hernia repair at POD 2 [14]. This is supported by a meta-analysis by Ossola et al. [28] in 281 patients who found a highly significant reduction in pain in patients with AB compared to patients without AB on POD 1 and 5 (*p* = 0.001 and <0.001, respectively).

**TABLE 3:** Effect of wearing an abdominal binder (AB) on postoperative pain.

Study	Type of study	Population	Effect on pain
Paasch et al. [14]	Multi-centre randomised study 2 groups: 18 with an AB for 2 weeks during the day 19 without an AB	Patients undergoing laparoscopic hernia surgery	It would appear that there is less pain in the belt group but this is only significant on post operative day (POD) 2 (*p* = 0.042)
Clay et al. [15]	Single-centre prospective randomised study 48 patients (23 with an AB and 25 without)	Patients undergoing a midline laparotomy of at least 12 cm wearing an AB during hospitalisation	Significantly less pain on 5th POD in the AB group (*p* = 0.004)
Christoffersen et al. [12]	Prospective randomised study between October 2012 and September 2013. 56 patients: 28 with AB and 28 without	Wearing the AB for 7 full days postoperatively for an umbilical or epigastric hernia or hernia between 2 and 6 cm by laparoscopy	No difference
Di Mascio et al. [30]	Systematic review and meta-analysis aiming to define the effectiveness of an AB on pain and psychological distress in patients after caesarean section. 4 articles 601 patients: 310 with AB and 291 without	Patients after caesarean section	No difference in pain. Pain measurement by VAS
Paasch et al. [25]	Questionnaire sent to 270 operated-on patients to define postoperative recommendations	Patients undergoing incisional hernia surgery (open and minimally invasive) Wearing the support belt for 4–6 weeks during the day	163 AB-wearing patients responded. 115 patients reported feeling a reduction in pain
Rothman et al. [26]	Review of literature studying the wearing of AB 8 articles, 578 patients	Patients undergoing laparotomy, laparoscopy or abdominoplasty surgery	No significant results
Ossola et al. [28]	Meta-analysis aiming to assess the efficacy of AB for postoperative pain with 5 randomised controlled trials. 281 patients randomised in 2 groups (AB vs. non-AB)	Patients who had a midline laparotomy	Significantly less pain on POD 1 (*p* = 0.01) and POD 5 (*p* < 0.01)

POD, postoperative day.

### Surgical-Site Occurrences (SSO) (Table 4)

Studies focusing on the effect of abdominal support on SSO have often been performed after hernia surgery. A study describing the technique of laparoscopic intraperitoneal mesh placement in 2000 [23] reported that the rate of postoperative seroma was lower after the prescription of an AB (32% vs. 18%).

**TABLE 4:** Effect of abdominal binders (AB) on the occurrence of postoperative seroma.

Study	Type of study	Population	Effect on seromas
Chowbey et al. [23]	Retrospective descriptive study AB prescribed for 1 week	Patients undergoing laparoscopic hernia surgery with intraperitoneal prothesis	Decrease in seromas from 32% of patients to 18% No statistics
Ortiz et al. [13]	National prospective randomised multi-centre study on 40 patients [2 groups: AB for 2 weeks (21 patients) during the day or not wearing an AB (19 patients)]	Patient operated on for an incisional hernia using the Stoppa-Rives technique	Significantly more seroma in the belt group 13 vs. 9, *p* = 0.012 (main effect time)
Rothman et al. [26]	Literature review	Patients undergoing major or minor laparotomy, laparoscopy or abdominoplasty	No significant difference in the occurrence of seroma

There was no significant difference between the two groups in terms of pain at rest and impaired mobility. Patients in the AB group had a higher rate and larger seroma at day 14 (66.6% vs. 50%). However, the AB group had a significantly lower rate of surgical site infection at day 14 (4.8% vs. 27.8%, *p* = 0.004) [13]. The studies did not find any side effects attributed to the AB. No difference in respiratory function [15, 28] or abdominal pressure [26].

### Other Issues of Abdominal Support

Regarding return to activity, the only randomized trial [18] that found a significant result highlights a better walking distance in patients wearing an AB on postoperative day 5 (60 patients: 30 with and 30 without harness). In addition, 2 studies asked patients about their emotional state using a patient questionnaire. One of these studies [25] conducted on 163 patients, showed that 50 patients felt that the AB reduced their mobility and 115 felt that the AB reduced their pain. The other study [27] of 67 patients found that 64% experienced a subjective improvement in their symptoms (combining pain, heaviness, embarrassment, difficulty dressing, difficulty moving, difficulty breathing) and 97% experienced discomfort (including heat, friction, pain, itching).

Regarding postoperative discomfort, several studies of patients undergoing caesarean section show reduced discomfort in patients in the immediate postpartum period. 3 gynaecologic studies (Table 5) showed an improvement in the SDS (Symptom Distress Scale, calculated by collecting elements of nausea, vomiting, pain, anorexia, sleep disturbance, fatigue, difficulty breathing, cough, crying, restlessness, difficulty concentrating, body temperature, transit, and physical appearance). The first, by Karaca et al. [16] in a prospective randomized trial of 89 patients (45 with and 44 without a support belt), reported a better SDS score in the AB group at 8 h (*p* = 0.024), 24 h (*p* < 0.001), and 48 h (*p* < 0.001) after surgery. Another study by Ghana et al. [17] after randomizing 178 patients (89 with belt and 89 without) reported a significant difference in SDS in favour of the AB at 24 and 48 h with *p* < 0.001. Finally, a meta-analysis by Abd-ElGawad et al. [29] also found an improvement in SDS with the AB at 24 h (*p* < 0.001) and 48 h (*p* = 0.002). However, there are no data on symptoms at 48 h.

**TABLE 5:** Effect of wearing a abdominal binder (AB) on patient discomfort.

Study	Type of study	Population	Effect on discomfort
Karaca et al. [16]	Prospective randomised study between September 2017 and January 2018	89 patients undergoing caesarean section with randomised selection of 45 patients with AB and 44 without AB	SDS measurement: In favour of the AB 8 h after the caesarean: 11.8 vs. 12.9 (*p* = 0.024) 24 h after, 10.7 vs. 13.6 (*p* < 0.001). 48 h after, 8.8 vs. 12.6 (*p* < 0.001)
Ghana et al. [17]	Prospective randomised study between January and October 2015	178 patients operated on (89 with AB and 89 without AB)	Significantly lower SDS in favour of wearing an AB after 24 h (15 vs. 18, *p* < 0.001) and after 48 h (14 vs. 16, *p* < 0.001)
Abd-ElGawad et al. [29]	Meta-analysis	6 randomised trials with 702 patients (369 with AB and 333 without AB)	Improvement in SDS in the AB group with a reduction in the score of 1.87 (*p* = 0.001) at 24 h after the caesarean section and at 48 h after (1.87, *p* = 0.002)

SDS, symptom distress scale.

## Discussion

Current evidence on the effects of AB does not support its use after any abdominal surgery: gastrointestinal, gynaecologic, urologic, or plastic. There is little or no evidence of the benefit of an AB in reducing complications and/or recurrence. Prescribing guidelines for these supports do not include specific recommendations on how the support should be worn: daytime, day and nighttime, during exercise, or for how long the belt should be worn [11, 24]. Some data from small randomized trials suggest a reduction in pain during the first postoperative days [13, 15]. The most recent meta-analysis by Ossola et al. [28] also shows that there was significantly less pain in the AB group at the first and fifth PODs and that there was also an increase in physical activity from the fourth POD. Finally, in their meta-analysis, the authors did not find any side effects of wearing the AB. However, due to the small number of RCTs, they also concluded that the evidence for wearing an AB postoperatively is weak and that routine wearing of an AB should not be recommended. Therefore, it does not appear necessary to recommend wearing an AB for more than 1 week.

Regarding the intuitive belief that reduced tension at the time of laparotomy leads to a lower incidence of incisional after laparotomy or fewer recurrences after incisional hernia repair, there is no evidence to support such a practice. Furthermore, the natural history of incisional hernia and incisional hernia recurrence suggests that these events most commonly occur within 18–24 months [4, 5] after abdominal surgery, well beyond the time limit for wearing the support belt.

One of the benefits would be the subjective reassurance of the patient postoperatively, which is difficult to determine as it is so subjective [25, 27]. However, given the paucity of objective evidence and the cost of an AB, it seems difficult to justify a recommendation to wear an AB after any abdominal surgery or even after any laparotomy. This is reaffirmed by the American and European guidelines in 2022 [31] which state: “No recommendation can be made for or against the use of postoperative binders owing to the lack of data on their effect on incisional hernia or burst abdomen.”

However, no harmful effect of this support belt in the postoperative period has been demonstrated [15, 26, 28]. It could lead to a faster return to activity [18], which in turn could have an economic impact due to reduced hospitalization time and a faster return to work.

## Conclusion

Based on this literature review, it seems reasonable to conduct a large, multi-centre, prospective, randomized trial to assess the medical and socio-economic effects of wearing an AB postoperatively.

## References

[1] Sauerland S Walgenbach M Habermalz B Seiler CM Miserez M. Laparoscopic versus Open Surgical Techniques for Ventral or Incisional Hernia Repair. Cochrane Database Syst Rev (2011)(3) CD007781. 10.1002/14651858.CD007781.pub2 21412910 PMC13389912

[2] Eker HH Hansson BM Buunen M Janssen IM Pierik RE Hop WC Laparoscopic vs. Open Incisional Hernia Repair: A Randomized Clinical Trial. JAMA Surg (2013) 148(3):259–63. PMID: 23552714. 10.1001/jamasurg.2013.1466 23552714

[3] Stabilini C Bracale U Pignata G Frascio M Casaccia M Pelosi P Laparoscopic Bridging vs. Anatomic Open Reconstruction for Midline Abdominal Hernia Mesh Repair [LABOR]: Single-Blinded, Multicenter, Randomized, Controlled Trial on Long-Term Functional Results. Trials (2013) 14:357. PMID: 24165473; PMCID: PMC4231609. 10.1186/1745-6215-14-357 24165473 PMC4231609

[4] Smith L Wilkes E Rolfe C Westlake P Cornish J Brooks P Incidence, Healthcare Resource Use and Costs Associated With Incisional Hernia Repair. J Abdom Wall Surg (2024) 3:12452. 10.3389/jaws.2024.12452 38481877 PMC10936754

[5] Ortega-Deballon P Renard Y de Launay J Lafon T Roset Q Passot G. Incidence, Risk Factors, and Burden of Incisional Hernia Repair After Abdominal Surgery in France: A Nationwide Study. Hernia (2023) 27(4):861–71. Epub 2023 Jun 27. PMID: 37368183; PMCID: PMC10374769. 10.1007/s10029-023-02825-9 37368183 PMC10374769

[6] Le Huu NR Mege D Ouaïssi M Sielezneff I Sastre B. Incidence and Prevention of Ventral Incisional Hernia. J Visc Surg (2012) 149(5 Suppl. l):e3–14. Epub 2012 Nov 9. PMID: 23142402. 10.1016/j.jviscsurg.2012.05.004 23142402

[7] Liang MK Subramanian A Awad SS. Laparoscopic Transcutaneous Closure of Central Defects in Laparoscopic Incisional Hernia Repair. Surg Laparosc Endosc Percutan Tech (2012) 22(2):e66–70. PMID: 22487642. 10.1097/SLE.0b013e3182471fd2 22487642

[8] Chelala E Baraké H Estievenart J Dessily M Charara F Allé JL. Long-Term Outcomes of 1326 Laparoscopic Incisional and Ventral Hernia Repair With the Routine Suturing Concept: ASingle Institution Experience. Hernia (2016) 20(1):101–10. Epub 2015 Jun 21. PMID: 26093891. 10.1007/s10029-015-1397-y 26093891

[9] Gillion J-F Ortega-Deballon P. Éventrations Postopératoires: Rapport Présenté au 121e Congrès Français de Chirurgie, Paris, 15-17 mai 2019. Montrouge: Arnette (2019).

[10] Gignoux B Bayon Y Martin D Phan R Augusto V Darnis B Incidence and Risk Factors for Incisional Hernia and Recurrence: Retrospective Analysis of the French National Database. Colorectal Dis (2021) 23(6):1515–23. Epub 2021 Apr 5. PMID: 33570808. 10.1111/codi.15581 33570808

[11] Bouvier A Rat P Drissi-Chbihi F Bonnetain F Lacaine F Mariette C Abdominal Binders After Laparotomy: Review of the Literature and French Survey of Policies. Hernia (2014) 18(4):501–6. Epub 2014 May 17. PMID: 24838292. 10.1007/s10029-014-1264-2 24838292

[12] Christoffersen MW Olsen BH Rosenberg J Bisgaard T. Randomized Clinical Trial on the Postoperative Use of an Abdominal Binder After Laparoscopic Umbilical and Epigastric Hernia Repair. Hernia (2015) 19(1):147–53. Epub 2014 Sep 9. PMID: 25201555. 10.1007/s10029-014-1289-6 25201555

[13] Ortiz PR Lorenz E Meyer F Croner R Lünse S Hunger R The Effect of an Abdominal Binder on Postoperative Outcome After Open Incisional Hernia Repair in Sublay Technique: A Multicenter, Randomized Pilot Trial (ABIHR-II). Hernia (2023) 27(5):1263–71. Epub 2023 Jul 19. PMID: 37466732; PMCID: PMC10533646. 10.1007/s10029-023-02838-4 37466732 PMC10533646

[14] Paasch C Santo G Aljedani N Ortiz P Bruckert L Hünerbein M The Effect of an Abdominal Binder on Postoperative Pain After Laparoscopic Incisional Hernia Repair–A Multicenter, Randomized Pilot Trial (ABIHR-I) of the Intraperitoneal Onlay-Mesh Technique. Dtsch Arztebl Int (2021) 118(37):607–13. Epub 2021 Sep 24. PMID: 34857076; PMCID: PMC8704821. 10.3238/arztebl.m2021.0250 34857076 PMC8704821

[15] Clay L Gunnarsson U Franklin KA Strigård K. Effect of an Elastic Girdle on Lung Function, Intra-Abdominal Pressure, and Pain After Midline Laparotomy: A Randomized Controlled Trial. Int J Colorectal Dis (2014) 29(6):715–21. Epub 2014 Jan 28. PMID: 24468797. 10.1007/s00384-014-1834-x 24468797

[16] Karaca I Ozturk M Alay I Ince O Karaca SY Erdogan VS Influence of Abdominal Binder Usage After Cesarean Delivery on Postoperative Mobilization, Pain and Distress: A Randomized Controlled Trial. Eurasian J Med (2019) 51(3):214–8. PMID: 31692751; PMCID: PMC6812913. 10.5152/eurasianjmed.2019.18457 31692751 PMC6812913

[17] Ghana S Hakimi S Mirghafourvand M Abbasalizadeh F Behnampour N. Randomized Controlled Trial of Abdominal Binders for Postoperative Pain, Distress, and Blood Loss After Cesarean Delivery. Int J Gynaecol Obstet (2017) 137(3):271–6. Epub 2017 Mar 28. PMID: 28241386. 10.1002/ijgo.12134 28241386

[18] Cheifetz O Lucy SD Overend TJ Crowe J. The Effect of Abdominal Support on Functional Outcomes in Patients Following Major Abdominal Surgery: A Randomized Controlled Trial. Physiother Can 2010 Summer (2010) 62(3):242–53. Epub 2010 Jul 23. PMID: 21629603; PMCID: PMC2909864. 10.3138/physio.62.3.242 PMC2909864 21629603

[19] Page MJ McKenzie JE Bossuyt PM Boutron I Hoffmann TC Mulrow CD The PRISMA 2020 Statement: An Updated Guideline for Reporting Systematic Reviews. BMJ (2021) 372:n71. PMID: 33782057; PMCID: PMC8005924. 10.1136/bmj.n71 33782057 PMC8005924

[20] Hsiao WC Young KC Wang ST Lin PW. Incisional Hernia After Laparotomy: Prospective Randomized Comparison Between Early-Absorbable and Late-Absorbable Suture Materials. World J Surg (2000) 24(6):747–51. discussion 752, PMID: 10773130. 10.1007/s002689910120 10773130

[21] Sterne JA Hernán MA Reeves BC Savović J Berkman ND Viswanathan M ROBINS-I: A Tool for Assessing Risk of Bias in Non-Randomised Studies of Interventions. BMJ (2016) 355:i4919. PMID: 27733354; PMCID: PMC5062054. 10.1136/bmj.i4919 27733354 PMC5062054

[22] Guyatt G Oxman AD Akl EA Kunz R Vist G Brozek J GRADE Guidelines: 1. Introduction-GRADE Evidence Profiles and Summary of Findings Tables. J Clin Epidemiol (2011) 64(4):383–94. Epub 2010 Dec 31. PMID: 21195583. 10.1016/j.jclinepi.2010.04.026 21195583

[23] Chowbey PK Sharma A Khullar R Mann V Baijal M Vashistha A. Laparoscopic Ventral Hernia Repair. J Laparoendosc Adv Surg Tech A (2000) 10(2):79–84. PMID: 10794211. 10.1089/lap.2000.10.79 10794211

[24] Paasch C Anders S Strik MW. Postoperative-Treatment Following Open Incisional Hernia Repair: A Survey and a Review of Literature. Int J Surg (2018) 53:320–5. Epub 2018 Apr 12. PMID: 29656131. 10.1016/j.ijsu.2018.04.014 29656131

[25] Paasch C Lorenz E Anders S De Santo G Boettge K Gauger U Patient Reported Outcome Following Incisional Hernia Repair: A Survey on 163 Patients at Two Maximum Care Hospitals. Ann Med Surg (Lond) (2019) 44:5–12. PMID: 31249685; PMCID: PMC6586918. 10.1016/j.amsu.2019.06.005 31249685 PMC6586918

[26] Rothman JP Gunnarsson U Bisgaard T. Abdominal Binders May Reduce Pain and Improve Physical Function After Major Abdominal Surgery - A Systematic Review. Dan Med J (2014) 61(11):A4941. PMID: 25370959. 25370959

[27] Strigård K Stark B Bogren A Gunnarsson U. Ventral Hernia and Patient Experience of an Elastic Girdle. ANZ J Surg (2015) 85(7-8):525–8. Epub 2014 Dec 5. PMID: 25475523. 10.1111/ans.12924 25475523

[28] Ossola P Mascioli F Coletta D Pizzato M Bononi M. Evidence on Postoperative Abdominal Binding: A Systematic Review With Meta-Analysis of Randomized Controlled Trials. Surgeon (2021) 19(4):244–51. Epub 2020 Aug 6. PMID: 32773235. 10.1016/j.surge.2020.07.003 32773235

[29] Abd-ElGawad M Said Ali A Abdelmonem M Elshamy NH Abdeltawab AK Abd El-Shafea M The Effectiveness of the Abdominal Binder in Relieving Pain After Cesarean Delivery: A Systematic Review and Meta-Analysis of Randomized Controlled Trials. Int J Gynaecol Obstet (2021) 154(1):7–16. Epub 2021 Feb 26. PMID: 33471362. 10.1002/ijgo.13607 33471362

[30] Di Mascio D Caruso G Prata G Saccone G Terrin G Giancotti A The Efficacy of Abdominal Binders in Reducing Postoperative Pain and Distress After Cesarean Delivery: A Meta-Analysis of Randomized Controlled Trials. Eur J Obstet Gynecol Reprod Biol (2021) 262:73–9. Epub 2021 May 9. PMID: 33993065. 10.1016/j.ejogrb.2021.05.014 33993065

[31] Deerenberg EB Henriksen NA Antoniou GA Antoniou SA Bramer WM Fischer JP Updated Guideline for Closure of Abdominal Wall Incisions From the European and American Hernia Societies. Br J Surg (2022) 109(12):1239–50. Erratum in: Br J Surg. 2023 Jan 10;110(2):287. 10.1093/bjs/znac412. PMID: 36026550; PMCID: PMC10364727. 10.1093/bjs/znac302 36026550 PMC10364727

